# Azithromycin versus placebo for the treatment of HIV-associated chronic lung disease in children and adolescents (BREATHE trial): study protocol for a randomised controlled trial

**DOI:** 10.1186/s13063-017-2344-2

**Published:** 2017-12-28

**Authors:** Carmen Gonzalez-Martinez, Katharina Kranzer, Grace McHugh, Elizabeth L. Corbett, Hilda Mujuru, Mark P. Nicol, Sarah Rowland-Jones, Andrea M. Rehman, Tore J. Gutteberg, Trond Flaegstad, Jon O. Odland, Rashida A. Ferrand, the BREATHE study team, Tsitsi Bandason, Pauline Cavanagh, Ethel Dauya, Edith Majonga, Beauty Makamure, Gugulethu Newton Mapurisa, Slee Mbhele, Brewster Wisdom Moyo, Lucky Gift Ngwira, Jamie Rylance, Victoria Simms, Evgeniya Sovershaeva, Helen Anne Weiss, Louis-Marie Yindom

**Affiliations:** 1 Liverpool School of Tropical Medicine, Pembroke Place, Liverpool, L3 5QA UK; 2 Malawi-Liverpool Wellcome Trust Clinical Research Programme, PO Box 30096, Chichiri, Blantyre 3, Malawi; 3 Department of Paediatrics and Child Health, College of Medicine, University of Malawi, Private Bag 360, Chichiri, Blantyre 3, Malawi; 4 Department of Clinical Research, London School of Hygiene and Tropical Medicine, Keppel Street, London, WC1E 7HT UK; 5 Biomedical Research and Training Institute, 10 Seagrave Road, Harare, Zimbabwe; 6 Department of Infectious Disease Epidemiology, London School of Hygiene and Tropical Medicine, Keppel Street, London, WC1E 7HT UK; 7 Department of Paediatrics, University of Zimbabwe, PO Box A178, Avondale, Harare, Zimbabwe; 8 Division of Clinical Microbiology, University of Cape Town, Anzio Road, Cape Town, South Africa; 9 National Health Laboratory Service, Johannesburg, South Africa; 10 Nuffield Department of Medicine, Old Road Campus, University of Oxford, Roosevelt Drive, Oxford, OX3 7FZ UK; 11 MRC Tropical Epidemiology Group, London School of Hygiene and Tropical Medicine, Keppel Street, London, WC1E 7HT UK; 12 Department of Microbiology and Infection Control, University Hospital of North Norway, N-9038 Tromsø, Norway; 13 Faculty of Health Sciences, Arctic University of Norway, N-9037 Tromsø, Norway; 14 Department of Paediatrics, University Hospital of North Norway, N-9038 Tromsø, Norway; 15 School of Health Systems and Public Health, Faculty of Health Sciences, University of Pretoria, Hatfield, South Africa

**Keywords:** Chronic lung disease, Azithromycin, HIV, FEV_1_, Africa, Children, Obliterative bronchiolitis

## Abstract

**Background:**

Human immunodeficiency virus (HIV)-related chronic lung disease (CLD) among children is associated with substantial morbidity, despite antiretroviral therapy. This may be a consequence of repeated respiratory tract infections and/or dysregulated immune activation that accompanies HIV infection. Macrolides have anti-inflammatory and antimicrobial properties, and we hypothesised that azithromycin would reduce decline in lung function and morbidity through preventing respiratory tract infections and controlling systemic inflammation.

**Methods/design:**

We are conducting a multicentre (Malawi and Zimbabwe), double-blind, randomised controlled trial of a 12-month course of weekly azithromycin versus placebo. The primary outcome is the mean change in forced expiratory volume in 1 second (FEV_1_) z-score at 12 months. Participants are followed up to 18 months to explore the durability of effect. Secondary outcomes are FEV_1_ z-score at 18 months, time to death, time to first acute respiratory exacerbation, number of exacerbations, number of hospitalisations, weight for age z-score at 12 and 18 months, number of adverse events, number of malaria episodes, number of bloodstream *Salmonella* typhi infections and number of gastroenteritis episodes. Participants will be followed up 3-monthly, and lung function will be assessed every 6 months. Laboratory substudies will be done to investigate the impact of azithromycin on systemic inflammation and on development of antimicrobial resistance as well as impact on the nasopharyngeal, lung and gut microbiome.

**Discussion:**

The results of this trial will be of clinical relevance because there are no established guidelines on the treatment and management of HIV-associated CLD in children in sub-Saharan Africa, where 80% of the world’s HIV-infected children live and where HIV-associated CLD is highly prevalent.

**Trial registration:**

ClinicalTrials.gov, NCT02426112. Registered on 21 April 2015.

**Electronic supplementary material:**

The online version of this article (doi:10.1186/s13063-017-2344-2) contains supplementary material, which is available to authorized users.

## Background

Respiratory disease is the most common manifestation of human immunodeficiency virus (HIV)/acquired immunodeficiency syndrome (AIDS) among children in sub-Saharan Africa, accounting for more than 50% of HIV-associated mortality [1–8]. The use of antiretroviral therapy (ART) and co-trimoxazole prophylaxis has contributed to a reduction in the rate of acute respiratory tract infections and mortality among HIV-infected children in both high- and low-resource settings [9].

However, studies in recent years in sub-Saharan Africa have demonstrated that about 30% of perinatally HIV-infected older children and adolescents have chronic respiratory symptoms, including chronic cough, reduced exercise tolerance and significantly impaired lung function [4, 8]. In these studies, even participants with pronounced respiratory impairment looked well at rest, and plain radiological abnormalities were subtle. High-resolution computed tomography findings showed predominantly small airway disease consistent with constrictive obliterative bronchiolitis (OB) [4, 10]. Importantly, no association was observed between abnormal lung function, ART use or duration or CD4 count, suggesting that this form of HIV-related chronic lung disease may not be responsive to ART.

OB is a chronic obstructive lung disease that follows a severe insult to the lower respiratory tract, resulting in fibrosis of the small airways [11]. The most common presentation is the post-infectious variant, closely related to severe viral infection in the first 3 years of life [12]. It is also seen in the context of allogeneic haematopoietic stem cell (HSC) and lung transplant recipients as a result of a graft-versus-host reaction [13, 14]. HIV is associated with both high incidence of respiratory infections and persistent immune activation despite ART [15–17]. Thus, HIV-associated OB may share causal pathways with both post-infectious and post-transplant variants.

Evidence regarding the efficacy of treatment modalities in OB is sparse. Observational studies have shown some improvement of lung function in a small number of children treated with high-dose pulse corticosteroids for post-transplant OB [14, 18]. Studies in lung transplant-associated OB have shown a positive effect of azithromycin, whereas a small randomised controlled trial of patients with established HSC-associated OB failed to show an effect [19, 20]. Authors of case series including patients with post-infectious and post-transplant OB have reported a benefit of azithromycin on lung function and on rate of exacerbations [21].

Azithromycin is a macrolide antibiotic with bacteriostatic activity against the most common respiratory bacterial pathogens. However, it also has a robust immunomodulatory effect resulting in decreased production of pro-inflammatory cytokines in the acute phase and resolution of chronic inflammation in the later phases. Specifically, azithromycin has direct activity on airway epithelial cells to maintain their function and reduce mucus secretion [20, 22–24]. These characteristics have resulted in the use of azithromycin in the management of a variety of chronic lung diseases, including cystic fibrosis [25], non-cystic fibrosis bronchiectasis [26], bronchiolitis obliterans syndrome [19, 27] and chronic obstructive pulmonary disease [28]. The intracellular uptake of azithromycin is high, and hepatic excretion is slow, resulting in a long half-life that enables infrequent dosing. We will test the hypothesis that prophylactic azithromycin is effective, through its antimicrobial and anti-inflammatory properties, in preventing worsening of lung function and in reducing exacerbations in children and adolescents receiving ART who have HIV-associated chronic lung disease.

## Methods/design

### Study design

BREATHE (Bronchopulmonary function in response to azithromycin treatment for chronic lung disease in HIV-infected children) is a two-site, double-blind, placebo-controlled, individually randomised trial in which we intend to enrol 400 perinatally HIV-infected children and adolescents aged 6–19 years with HIV-associated chronic lung disease who have been receiving ART for a minimum of 6 months. Participants will be enrolled from the outpatient HIV clinics in Harare, Zimbabwe, and Blantyre, Malawi.

### Study intervention

Participants are randomly assigned to receive either azithromycin or placebo in a 1:1 ratio. Weight-band azithromycin (10–19.9 kg, 250 mg; 20–29.9 kg, 500 mg; 30–39.9 kg, 750 mg; > 40 kg, 1250 mg/week) or placebo will be given weekly under direct observation by a treatment monitor identified within the family for a total of 12 months.

### Study population

HIV-infected children and adolescents with HIV-associated chronic lung disease attending outpatient HIV clinics in Harare and Blantyre will be enrolled in the trial. Individuals aged 6–19 years will be approached together with their guardians during their routine HIV outpatient visits and provided with information about the study. Once informed consent is obtained for screening, eligibility will be established using a multi-step screening procedure. Chronic lung disease will be established by spirometry (forced expiratory volume in 1 second [FEV_1_] z-score less than −1.0) with no reversibility (< 12% improvement in FEV_1_ after salbutamol 200 μg inhaled using a spacer). Spirometry will be performed using the EasyOne™ spirometer (ndd Medical Technologies Inc., Andover, MA, USA) by trained research staff certified in performing spirometry and following the American Thoracic Society guidelines. Those who meet the criteria for chronic lung disease will undergo further tests, including a urine pregnancy test (for girls who have reached menarche), serum creatinine, alanine aminotransferase (ALT), an electrocardiogram and screening for tuberculosis (TB). For TB screening, we will use the Xpert™ MTB/RIF (Cepheid, Sunnyvale, CA, USA) to test one sputum sample obtained either spontaneously or through induction.

### Inclusion/exclusion criteria

Perinatally HIV-infected children and adolescents aged 6–19 years who have been receiving ART for at least 6 months for HIV-associated chronic lung disease, who have a firm home address and a stable guardian, and with consent from the guardian and assent from the participant (for those aged < 18 years; those aged ≥ 18 years to consent independently) will be eligible for inclusion. Exclusion criteria will include having a condition that may prove fatal during the study period (e.g., malignancy), TB or acute respiratory tract infection at the time of screening, pregnancy or breastfeeding, history of cardiac arrhythmia, a prolonged QTc interval, abnormal creatinine clearance or elevated ALT, known macrolide hypersensitivity, and concomitant use of digoxin and/or fluconazole (or other drugs known to prolong the QTc interval) (Table 1).

### Study procedures

Eligible individuals will be randomised to either azithromycin or placebo. The allocation ratio will be 1:1 by block randomisation with variable-length blocks stratified by site. The randomisation schedule and allocation list will be generated using Stata™ version 14 software (2015 release; StataCorp, College Station, TX, USA) by an independent statistician not connected with the study. The allocation list will be sent directly to the study pharmacists at both trial sites, who will prepare the study medication. Participants and study personnel will therefore be masked to treatment allocation. The pharmacist will be provided with only randomly allocated numbers assigning participants to arm 1 or arm 2, with each number linked to a study number, and therefore the pharmacist will also remain blinded. It will not be possible to have pre-prepared medication packs, owing to changes of dose with weight.

**Fig. 1:**
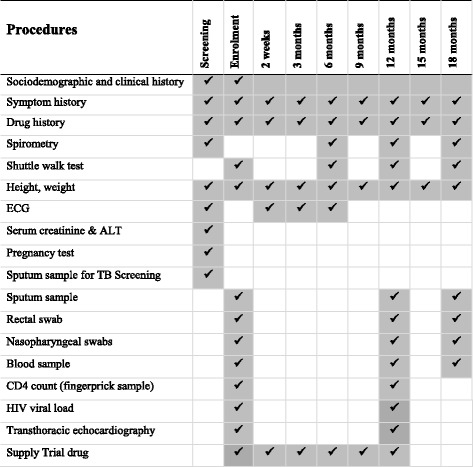
Schedule of trial procedures. *ALT* Alanine aminotransferase, *ECG* Echocardiography, *HIV* Human immunodeficiency virus, *TB* Tuberculosis

After randomisation, spirometry will be repeated, and cardiopulmonary fitness will be evaluated using the incremental shuttle walk test. Transthoracic echocardiography will be performed by a trained echocardiographer using the SonoSite™ M-Turbo echocardiography system (FUJIFILM SonoSite, Bothell, WA, USA) at the Malawi site and the Mindray™ DC-N6 echocardiography system (Mindray, Shenzhen, China) at the Zimbabwe site to assess right heart function and pulmonary hypertension. The research nurse will take a rectal swab, nasal aspirate, and sputum and blood samples. The blood sample will be used to measure CD4 count with the Pima™ Analyser (Alere, Orlando, FL, USA) and to perform viral load testing (Xpert™ HIV-1 Viral Load; Cepheid). The study drug will be dispensed by the study pharmacist. After the enrolment visit, follow-up visits will be scheduled at 2 weeks and 3-monthly thereafter. Participants will be followed for a minimum of 12 months to ascertain the primary outcome and for a further 6 months (i.e., 6 months following completion of study drug) to investigate durability of effect (if any) of the intervention. Procedures undertaken at the follow-up visits are summarised in Fig. 1.

Participants will be instructed to attend unscheduled visits if they develop acute symptoms. For specific acute symptoms, investigations and management will be as follows: for diarrhoeal episodes (Division of AIDS [DAIDS] severity grade ≥ 3), a rectal swab and *Clostridium difficile* rapid test will be taken (C. Diff Quick Check Complete™; Alere) and if positive will be treated with metronidazole for 7 days. For acute respiratory exacerbations, sputum and nasal swabs will be taken and will be treated with amoxicillin-clavulanate for 10 days; if there is no improvement, a chest radiograph and culture for TB will be obtained. For febrile episodes, blood cultures, malaria slides and or malaria rapid diagnostic tests and rectal swabs will be taken, and management will be carried out according to national guidelines. For other acute symptoms, investigations and management will be carried out at the discretion of the treating clinician. A nasal swab, rectal swab, and sputum and blood samples will be collected at study visits for measurement of immune activation markers, investigation of antimicrobial resistance and assessment of changes in the gut and respiratory microbiome.

### Risks

Potential risks include an allergic reaction or adverse reaction to the medication or placebo. Examples of potential side effects include nausea, vomiting, diarrhoea and skin reactions. Each of these risks and any other unexpected outcomes will be monitored at study visits. Criteria for stopping the study drug are allergic reaction, QTc prolongation > 500 milliseconds, hepatic toxicity with ALT > 80 IU/L, concomitant use of drugs known to produce QTc prolongation, pregnancy during the course of the study and any adverse reaction with DAIDS severity grade > 3. In case of any serious adverse events, the institutional review boards and the data and monitoring safety board (DSMB) will be notified. Unblinding of treating physicians will be carried out in case of pregnancy or other events after decision by the DSMB.

### Outcome measures

The primary outcome is the mean difference between trial arms in FEV_1_ z-scores (generated using Global Lung Function Initiative reference standards) between trial arms at 12 months after initiation of the study drug, adjusted for site and baseline FEV_1_ z-score. The secondary outcomes are described in Table 2.

**Table 1:** Inclusion/exclusion criteria

Inclusion criteria • Perinatally acquired HIV (self-report of no sexual debut or blood transfusions, a history of orphanhood owing to maternal HIV disease and/or a history of sibling death due to HIV, and characteristic clinical features (i.e., stunting, history of recurrent minor infections in childhood and planar warts) • Aged 6–19 years • On first- or second-line ART for ≥ 6 months • Chronic lung disease (FEV_1_ z-score less than −1 and < 12% improvement with bronchodilator) • Firm home address accessible in Blantyre/Harare and intending to remain there for 18 months • Stable caregiver for participants aged < 18 years • HIV status disclosed to the child (for those aged > 12 years) • Informed consent to participate in the trial (for those aged < 18 years, consent from guardian and assent from participant; for those aged ≥ 18 years, consent from participant)
Exclusion criteria • Any condition that may prove fatal during the study period • Diagnosis of tuberculosis at enrolment • Acute respiratory tract infection during enrolment • Pregnancy and breastfeeding • History of prolonged QTc syndrome or current or planned therapy with drugs likely to cause cardiac dysrhythmias • History of cholestatic jaundice or hepatic dysfunction associated with previous use of azithromycin or known hypersensitivity to a macrolide or ketolide drug • Prolonged QTc interval (> 440 milliseconds in males; > 460 milliseconds in females) • Creatinine clearance < 30 ml/minute • ALT > 80 IU/L • Concomitant use of digoxin and/or fluconazole • Lack of understanding of the study procedure or uncooperative behaviour

*Abbreviations: ALT* Alanine aminotransferase, *ART* Antiretroviral therapy, *FEV*_*1*_ Forced expiratory volume in 1 second, *HIV* Human immunodeficiency virus

**Table 2:** Secondary outcomes

Outcome	Time point
Time to first respiratory exacerbation	
Time to death	
Number of acute exacerbations	By 12 months
Number of hospitalisations	By 12 months
Number of mild, moderate and severe adverse events	By 12 months
Mean weight-for-age z-score adjusted for baseline	By 12 months
Incidence of infectious episodes (*Salmonella* typhi, malaria, gastroenteritis)	By 12 months
Durability of effect (FEV_1_ z-score)	18 months

*FEV*_*1*_ Forced expiratory volume in 1 second

Other outcomes will be the effect of azithromycin on (1) diversity and composition of respiratory and gut microbiome of children with HIV-associated chronic lung disease, (2) antibiotic resistance of bacteria colonising the respiratory tract, and (3) biomarkers of systemic inflammation.

### Laboratory procedures

Nasopharyngeal and sputum samples will be frozen and stored at −80 °C for later batch processing, which will include routine bacterial culture for respiratory pathogens, antimicrobial susceptibility testing and 16S ribosomal RNA (rRNA) amplicon sequencing (microbiome analysis). Microbiome analysis of stool samples will also be done.

Archived plasma samples (isolated and stored at −80 °C) will be used to quantify the levels of soluble markers of immune activation and microbial translocation, including high-sensitivity C-reactive protein, d-dimers, interleukin-6, β_2_-microglobulin and soluble CD14 using multiplex bead assays (Luminex, Austin, TX, USA). Levels of bacterial 16S rRNA will be quantified using qRT-PCR to measure the extent of microbial translocation over time. The levels of these markers will be correlated with FEV_1_ z-scores and compared between trial arms.

### Data collection and management and analysis plan

Research nurses and assistants collect the data at baseline and follow-up and record it on electronic clinical record forms using Google Nexus™ tablets (Google, Mountain View, CA, USA) running OpenDataKit software. In both countries, data will be uploaded, processed and saved to a Microsoft Access database (Microsoft, Redmond, WA, USA) before being exported to Stata version 14.0 software and merged into a single database backed up on a regular basis. Consistency checks and checks for missing data are performed both at data entry and fortnightly once the database has been merged. Laboratory data will be recorded on paper forms and entered into the database using optical character recognition. Spirometric data will be uploaded directly from the spirometer. Echocardiographic data will be recorded on electronic clinical record forms, and these will be processed and saved to the Microsoft Access database for merging with the main trial database.

The primary analysis will be done on a modified intention-to-treat basis [29]. Secondary analyses will include a per-protocol analysis which will be defined using all adherence data prior to unblinding the study. Continuous outcomes will be compared between treatment groups, adjusting for site and baseline measures of the outcome and using linear regression to estimate the mean difference and corresponding 95% CI. Time-to-event outcomes will be assessed using Cox proportional hazards regression and graphically displayed using Kaplan-Meier estimates. Between-group comparisons of binary outcomes will be analysed with logistic regression to estimate ORs and 95% CIs. Count data (e.g., number of hospitalisations) will be analysed using Poisson regression to estimate incidence rate ratios and 95% CIs. All analyses will be adjusted for site. Pre-specified effect modification analyses will include site and baseline severity of lung disease.

### Sample size and power

The following assumptions were made to calculate the sample size:

- Mean FEV_1_ z-score of −2.04 in the control group
- Patients randomised in equal proportions to the two regimens
- Up to 25% of participants unassessable owing to loss to follow-up, death or suboptimal spirometric traces
- No change in FEV_1_ z-score in the control arm
- Difference in the trial arm mean FEV_1_ z-scores ranging from 0.15 to 0.3, an effect assumed to be of clinical relevance
- SD ranging from 0.55 to 0.82 to assess the impact of variability on the difference in mean value the study has power to detect

**Fig. 2:**
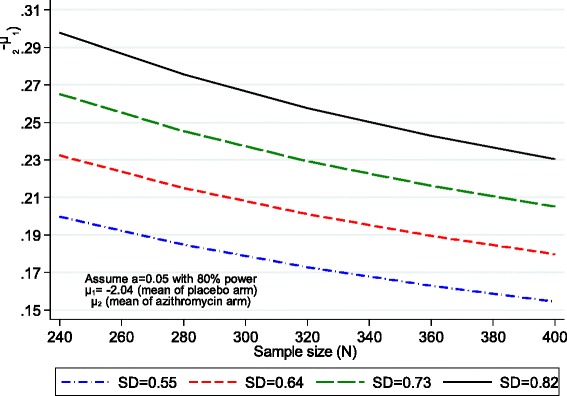
Sample size calculations. *FEV*_*1*_ Forced expiratory volume in 1 second

Under these assumptions, a sample size of 400 recruited participants and 300 participants with outcome data (25% unassessable based on previous studies of Zimbabwean children) will enable 80% power to detect a difference in trial arm means ranging between 0.17 and 0.23, an effect size (difference in means/SD) of 0.32 (Fig. 2).

### Ethics and regulatory bodies

The trial will be conducted in accordance with the principles of the Declaration of Helsinki, in compliance with the protocol approved by relevant ethics committees, and according to good clinical practice standards [30]. No change in the protocol will be implemented without the prior review and approval of the regulatory authorities, except where it may be necessary to eliminate an immediate hazard to the trial participants. Details concerning the enrolment process can be found in the Standard Protocol Items: Recommendations for Interventional Trials (SPIRIT) checklist (Additional file 1) [31].

The trial sponsor is the London School of Hygiene and Tropical Medicine (LSHTM). The trial will be monitored by the Clinical Trials Unit of the LSHTM and externally by the University of Zimbabwe Research Support Centre in Zimbabwe and the Malawi-Liverpool Wellcome Trust Clinical Research Programme Clinical Trials Unit in Malawi.

### Publication

Results of this trial will be published on completion in a peer-reviewed scientific journal. The funder will not be involved in the analysis or interpretation of the data. The full anonymised dataset will be made available no longer than 18 months after completion of the trial.

## Discussion

The massive scale-up of ART programmes globally has resulted in a dramatic improvement in survival, such that increasing numbers of children perinatally infected with HIV, many of whom would have died in early childhood without HIV treatment, are now reaching adolescence. Thus, attention needs to be focused on addressing the chronic complications of long-standing HIV infection prevalent among this cohort of older HIV-infected survivors. Studies in recent years have demonstrated a high burden of chronic lung disease in children with HIV in sub-Saharan Africa, even among those receiving ART and virologically suppressed. The urgency with which evidence-based management guidelines are needed is starkly apparent: Nearly one-third of older children have chronic respiratory symptoms, and in the absence of any alternative therapeutic strategies, repeated treatment for presumptive TB is the only treatment offered and often administered. Our group has investigated bronchodilators and short course, high-dose steroids in patients with chronic lung disease who are receiving ART and isoniazid preventive therapy, with no suggestions of benefit (personal communication, T. Mwalukomo).

Whereas in the pre-ART era, lymphoid interstitial pneumonitis (LIP) was the most common cause of chronic lung disease, studies in the ART era show that OB is the most likely underlying cause, with LIP being an exceptional finding. OB is a potentially life-threatening condition that can progress to hypoxic respiratory failure and cor pulmonale and may impair lung growth in children. OB results from small airway inflammation and subsequent fibrosis, and in the context of HIV, it is likely that inflammation is a consequence of both HIV-mediated chronic immune activation, resulting in end-organ damage, as well as repeated infections due to HIV-mediated immunodeficiency. We therefore postulate that azithromycin is a strong candidate as a therapeutic agent for HIV-associated chronic lung disease, given its broad-spectrum antibiotic activity and its anti-inflammatory and immunomodulatory properties, as well as demonstrated activity in similar chronic lung diseases and a good safety profile and tolerability. In this trial, we will investigate the effect of azithromycin on lung function as well as on acute exacerbations and other infections, and the trial will provide insight into the pathogenesis of this condition.

## Trial status

The current protocol is version 2.2, dated 21 August 2017. Enrolment started in June 2016 and will continue until June 2018.

## Additional file

Additional file 1: SPIRIT checklist. (DOC 147 kb)

## Abbreviations

AIDS: Acquired immunodeficiency syndrome
ALT: Alanine aminotransferase
ART: Antiretroviral therapy
BREATHE: Bronchopulmonary function in response to azithromycin treatment for chronic lung disease in HIV-infected children and adolescents trial
CLD: Chronic lung disease
DAIDS: Division of AIDS
DSMB: Data and safety monitoring board
ECG: Echocardiography
FEV_1_: Forced expiratory volume in 1 second
HIV: Human immunodeficiency virus
HSC: Haematopoietic stem cell
LIP: Lymphoid interstitial pneumonitis
LSHTM: London School of Hygiene and Tropical Medicine
OB: Obliterative bronchiolitis
rRNA: Ribosomal RNA
SPIRIT: Standard Protocol Items: Recommendations for Interventional Trials
TB: Tuberculosis
