# Supplementary material for: Azithromycin versus placebo for the treatment of HIV-associated chronic lung disease in children and adolescents (BREATHE trial): study protocol for a randomised controlled trial
Source: Trials. 2017 Dec 28;18:622. doi: 10.1186/s13063-017-2344-2 (PMC5745989; doi:10.1186/s13063-017-2344-2)
Supplement: Additional file 1: — SPIRIT checklist. (DOC 147 kb) [file 13063_2017_2344_MOESM1_ESM.doc]

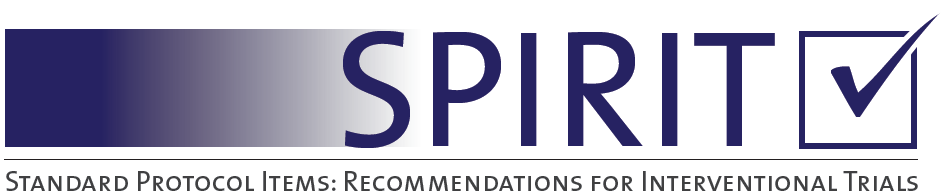


SPIRIT 2013 Checklist: Recommended items to address in a clinical trial protocol and related documents*

| Section/item | ItemNo | Description |
| --- | --- | --- |
| **Administrative information** | | |
| Title | 1 | **Bronchopulmonary function in response to Azithromycin treatment for chronic lung disease in HIV-infected children (BREATHE)** |
| Trial registration | 2a | **Trial registry name:** Clinical Trials.gov. **Number:** NCT02426112. **Date registered:** 21 Apr 2015 |
|  | 2b | **World Health Organisation Trial Registration Dataset**: See attached Table (Additional file 2) |
| Protocol version | 3 | **Protocol:** version 2.1; 30^th^ January 2017  **Revision chronology**  Version 1.2, 15 Dec 2015  Version 1.3, 23 Jun 2016  Version 1.4, 19 Aug 2016  Version 2, 26 Aug 2016  Primary reason for amendment: change in inclusion criteria (versions 1.2, 1.3, 1.4) and change in the definition of the primary outcome (version 2). |
| Funding | 4 | Funder: Medical Research Council of Norway. |
| Roles and responsibilities | 5a | **Names, affiliations, and roles of protocol contributors**  Carmen Gonzalez-Martinez^1,2,3^, Katharina Kranzer^4^, Grace McHugh^5^, Elizabeth L Corbett^2,6^, Hilda Mujuru^7^, Mark P Nicol^8^, Sarah Rowland-Jones^9^, Andrea M Rehman^10^, Tore J Gutteberg^11,12^, Trond Flaegstad^12,13^, Jon O Odland^12,14^, Rashida A Ferrand^5,6^  ^1^Liverpool School of Tropical Medicine, Liverpool, UK  ^2^Malawi-Liverpool-Wellcome Trust Clinical Research Programme, Blantyre, Malawi  ^3^Department of Paediatrics and Child Health, College of Medicine, Blantyre, Malawi  ^4^Department of Clinical Research, London School of Hygiene and Tropical Medicine, London, UK  ^5^Biomedical Research and Training Institute, Harare, Zimbabwe  ^6^Department of Infectious Disease Epidemiology, London School of Hygiene and Tropical Medicine, London, UK  ^7^Department of Paediatrics, University of Zimbabwe, Harare, Zimbabwe  ^8^Division of Clinical Microbiology, University of Cape Town, South Africa and National Health Laboratory Service, South Africa.  ^9^University of Oxford, Oxford, UK  ^10^MRC Tropical Epidemiology Group, London School of Hygiene and Tropical Medicine, London, UK  ^11^Department of Microbiology and Infection Control, University Hospital of North Norway, romsø, Norway  ^12^Faculty of Health Sciences, the Arctic University of Norway,Tromsø, Norway  ^13^Department of Paediatrics, University Hospital of North Norway, Tromsø, Norway  ^14^School of Health Systems and Public Health, Faculty of Health Sciences, University of Pretoria, South Africa  **Corresponding author***  Carmen Gonzalez-Martinez  Malawi Liverpool Wellcome Trust Clinical Research Programme  P.O Box 30096  Chichiri  Blantyre 3  Carmen.Gonzalez@lstmed.ac.uk  +265 (0) 1876444  **Protocol contributors**  Grant holder: JOO  Conceived of the study: RAF, ELC  Protocol development: CGM, KK, GM, HM, MN, SRJ  Provided statistical expertise in trial design: HAW  Implemented the trial: CGM, GM, RAF  All authors contributed to refinement of the study protocol and approved the final manuscript. |
|  | 5b | The trial sponsor is London School of Hygiene & Tropical Medicine (Ref, No.: QA698), WC1E 7HT, Keppel Street, [+44 20 7636 8636](tel:%2B44 20 7636 8636), London, United Kingdom. Contact person: [patricia.henley@lshtm.ac.uk](mailto:patricia.henley@lshtm.ac.uk). |
|  | 5c | The funder and the sponsor played no role in the study design; collection, management, analysis, and interpretation of data; writing of the report; and the decision to submit the report for publication. |
|  | 5d | **Trial Steering Committee**   - Members: Prof Elizabeth Molyneux (College of Medicine, Blantyre, Malawi), Prof Heather Zar (University of Cape Town, South Africa), Dr Lynda Stranix-Chibanda (University of Zimbabwe), Prof Catherine Hankins (London School of Hygiene and Tropical Medicine, UK), Ms Amanda Madyadi (University of Zimbabwe). - Agreement of final protocol. - Recruitment of study participants. - Reviewing study progress and agreeing changes to the protocol if necessary. - Reviewing adverse events. - Making final decision to drop arms following recommendation from Data Safety and Monitoring Board (DSMB) recommendation.   **Data Safety and Monitoring Board**   - Independent members from the trial investigators. Prof Christopher Whitty (Chair), London School Tropical Medicine, UK; Prof Sarah Walker, University College London, UK; Prof Steven Graham, University of Melbourne, Australia. - Reviewing interim analysis results including safety data. - Unblinding. - Recommending that trial arms be dropped.   **Independent statistician**   - Dr Stephen Nash (London School of Hygiene and Tropical Medicine). - Responsible for randomisation and code breaking. |
| Introduction |  |  |
| Background and rationale | 6a | **Research question:** Does weekly Azithromycin preventive therapy improve lung function in HIV-infected children with chronic lung disease?  Despite efforts to eliminate mother to child transmission, 240,000 infants were newly infected with HIV at the end of 2013 globally, and the coverage of antiretroviral therapy (ART) in children was only 24%. Thus, HIV will continue to place a heavy burden on paediatric clinical services in sub-Saharan Africa, where 90% of the world’s HIV-infected children live. Hitherto, the main focus of HIV programs has been on meeting the need for massive scale-up of paediatric ART and on improving infant survival. Now, attention needs to shift to the growing cohort of older HIV-infected survivors, a group that has been almost entirely neglected until the last few years. If our finding, that over a third of older African children in HIV care have CLD, is also applicable to children growing up in the era of early infant diagnosis and care, then the urgency with which evidence-based management guidelines are needed becomes starkly apparent: this is a very common condition for which no evidence base exists to guide management. Our group has investigated bronchodilators, and short course, high-dose, steroids in patients with CLD who are on ART and isoniazid preventive therapy, with no suggestions of benefit. Unlike in younger age groups, CLD in older HIV-infected children does not appear to respond to ART. Proceeding to a Phase III RCT of azithromycin to treat CLD in children living with HIV is justified because of the need for prolonged ART treatment before benefit is anticipated, the minimal potential for harm, and the unusually strong *a priori* case for likely benefit from azithromycin as shown in similar conditions, such as cystic fibrosis, non-cystic fibrosis bronchiectasis and post lung transplantation obliterative bronchiolitis. |
|  | 6b | **Comparator:** Placebo |
| Objectives | 7 | Primary objective   1. To investigate whether adjuvant treatment with azithromycin results in improvement in lung function in HIV-infected children on ART with chronic lung disease.   Secondary objectives   1. To investigate the intervention effect on mortality and morbidity. 2. To investigate adverse events related to Azithromycin treatment. |
| Trial design | 8 | This is a Phase III individually randomised, double blind, trial of Azithromycin vs placebo. Allocation ratio will be 1:1 by block randomisation with variable length blocks stratified by site. |
| Methods: Participants, interventions, and outcomes | | |
| Study setting | 9 | The study will recruit in outpatient ART clinics in Blantyre (Malawi) and Harare (Zimbabwe). |
| Eligibility criteria | 10 | **Inclusion criteria**   1. Perinatally-acquired HIV 2. Age 6 to 19 years 3. On first or second line ART for at least six months 4. Chronic lung disease (FEV1 z-score < -1 & <12% improvement with bronchodilator) 5. Firm home address accessible in Blantyre/Harare and intending to remain there for 18 months 6. Stable caregiver for participants aged < 18 years 7. HIV status disclosed to the child (for those > 12 years) 8. Informed consent to participate in the trial (for < 18 years olds: consent from guardian and assent from participant; ≥ 18 years: consent from participant)   **Exclusion criteria**   1. Any condition that may prove fatal during the study period 2. Diagnosis of tuberculosis at enrolment 3. Acute respiratory tract infection during enrolment 4. Pregnancy and breastfeeding 5. History of prolonged QTc syndrome or current or planned therapy with drugs likely to cause cardiac dysrhythmias 6. History of cholestatic jaundice or hepatic dysfunction associated with previous use of azithromycin or known hypersensitivity to a macrolide or ketolide drug. 7. Prolonged QTc interval (>440milliseconds in males; >460milliseconds in females) 8. Creatinine clearance of <30 ml/minute 9. ALT > 80 IU/L 10. Concomitant use of digoxin and/or fluconazole 11. Lack of understanding of the study procedure or uncooperative behaviour |
| Interventions | 11a | Intervention arm 1: Azithromycin, 30 mg/kg weekly orally, for 12 months.  Intervention arm 2: Placebo, weekly orally, for 12 months. |
|  | 11b | There will be no change in dosing during the trial. Criteria for discontinuing the study drug will be: allergic reaction, QTc prolongation > 500 miliseconds, hepatic toxicity with ALT >80 IU/L, concomitant use of drugs known to produce QTc prolongation, pregnancy during the course of the study and any adverse reaction DAIDS > 3. |
|  | 11c | Participants will receive the study drug through directly observed therapy (DOTs) by a designated caregiver. Administration of the study drug will be reflected on a patient diary. The study team will count remaining pills at each study visit. |
|  | 11d | During the course of the study concomitant use of known drugs that prolong QTc interval will be avoided (especially long term use of Digoxin and Fluconazole, in other cases the study drug will be discontinued) |
| Outcomes | 12 | ***Primary and secondary outcomes*** Our primary outcome is the mean difference in FEV1 z-scores (generated using GLI reference standards) between trial arms at 12 months after initiation of the study drug, adjusted for site and baseline FEV1 z-score.  The secondary outcomes are time to first acute respiratory exacerbation, time to death, number of acute exacerbations and hospitalisations at 12 months, number and severity of adverse event at 12 months, weight gain measured as change in weight-for-age z-score adjusted for baseline at 12 and 18 months, number of Salmonella blood stream infections, malaria and gastroenteritis episodes at 12 months.  The durability of intervention effect will be established by comparing FEV1 z-scores at 18 months post-enrolment, six months after the study medication has been discontinued. Other outcomes will be the effect of azithromycin on i) diversity and composition of respiratory and gut microbiome of children with HIV-associated chronic lung disease; ii) antibiotic resistance of bacteria colonising the respiratory tract; and iii) biomarkers of systemic inflammation. |
| Participant timeline | 13 | The study drug is given at enrolment. First follow up visit will be at 2 weeks and thereafter every 3 months. The study drug will be stopped at 12 months and assessment of the primary outcome will be at 12 months spirometry. Participants will exit the study at 12-18 months. |
| Sample size | 14 | The following assumptions were made to calculate the sample size   - Mean FEV1 z-score of -2.50 among control group - Patients randomised in equal proportions to the two regimens - Up to 25% of participants un-assessable due loss to follow-up or death or suboptimal spirometric traces - No change in FEV1 z-score in the control arm - Difference in trial arm mean FEV1 z-scores ranging from 0.15 to 0.25, an effect assumed to be of clinical relevance - SD ranging from 0.54 to 0.71 to assess the impact of variability on the difference in mean we have power to detect   Under these assumptions a sample size of 400 recruited participants and 300 participants with outcome data (25% unassessable) will enable 80% power to detect a difference in trial arm means ranging between 0.17 and 0.23, an effect size (difference in means/SD) of 0.32. |
| Recruitment | 15 | Strategies for achieving adequate participant enrolment to reach target sample size include one-one information about the study and presence of the study team in as many outpatient clinics as possible. |
| **Methods: Assignment of interventions (for controlled trials)** | | |
| Allocation: |  |  |
| Sequence generation | 16a | The allocation sequence was obtained using computer-generated random numbers, Allocation ratio will be 1:1 by block randomisation with variable length blocks stratified by site. |
| Allocation concealment mechanism | 16b | Randomisation will be done by an independent statistician, the allocation list will be sent directly, by email, to the study pharmacists at both trial sites who will prepare the study medication. The pharmacist will only be provided with random allocated numbers allocating to arm 1 or 2 with each number linked to a study number, and therefore will also remain blinded. |
| Implementation | 16c | Dr Stephen Nash was responsible for randomisation and intervention assignment of trial units. |
| Blinding (masking) | 17a | This is a double blinded trial. |
|  | 17b | Unblinding will be allowed in case of pregnancy during the course of the study or in case of a serious event after instructions from the DSMB. |
| **Methods: Data collection, management, and analysis** | | |
| Data collection methods | 18a | All the data will be collected using open data kit (ODK) running on Nexus tablets and paper-based report forms processed through optical character recognition. In both countries data will be extracted, processed and saved into a Microsoft Access database, before being exported to Stata (version 14.0). Consistency checks and checks for missing data will be performed at data entry and after the database has been merged. |
|  | 18b | In order to retain participants and to complete follow-up of participants we plan to trace participants in the first 24 hours of missing an appointment, if contact is not possible by phone the study team will visit the participant’s home.  If the participants discontinues the study before 12 months will not be possible to measure the trial primary outcome. |
| Data management | 19 | Data will be managed primarily at the study sites by the site data managers. Data will be transferred on a weekly basis to the main data management hub at the London School of Hygiene and Tropical Medicine through a secured link. Data will be backed up daily at each site.  Source documents and paper based report forms will be stored in a locked filling cabinet separate from any participant’s identifier. |
| Statistical methods | 20a | The primary analysis will be modified intention-to-treat. Secondary analyses will include a per protocol analysis. The mean FEV1 z-score at 12 months (primary outcome) and at 18 months after treatment will be compared between treatment groups, adjusting for site and baseline FEV1 z-score, using linear regression to estimate the mean difference and corresponding 95% confidence interval (CI). Time-to-first exacerbation and time-to-death will be assessed using Cox proportional hazards regression and graphically displayed using Kaplan-Meier estimates. Between-group comparisons of binary outcomes will be analysed with logistic regression, to estimate odds ratios and 95% CI. Count data (e.g. number of hospitalisations) will be analysed using Poisson regression. All analyses will adjust for site. Continuous outcomes will be additionally adjusted for the baseline measure of the outcome. Missing outcome data will be imputed using multiple imputation, according to plausible assumptions about missingness. Pre-specified effect modification analyses will include site and baseline severity of lung disease. |
|  | 20b | Any variables that show imbalances will be adjusted for when analysing the trial outcomes at the end of the second stage using logistic regression with random effects. |
|  | 20c | Multiple imputation will be used, if needed, in case of missing data. |
| **Methods: Monitoring** | | |
| Data monitoring | 21a | Data Safety and Monitoring Board (guided by Damocles Charter)   - Independent members from the trial investigators. Prof Christopher Whitty (Chair), London School of Hygiene and Tropical Medicine; Prof Sarah Walker, University College London and Prof Steven Graham, University of Melbourne. - Performing interim analysis including safety data. |
|  | 21b | First interim analysis will be done after recruiting first 150 participants or earlier if there are any safety concerns. Criteria for stopping the trial will be futility or safety concerns in the intervention arm. |
| Harms | 22 | Potential risks include an allergic reaction or adverse reaction to the medication or placebo. Examples of potential side effects include nausea, vomiting, diarrhoea and skin reactions. Each of these risks and any other unexpected outcomes will be monitored at study visits. |
| Auditing | 23 | The trial will be monitored by the Clinical Trials Unit of the London School of Hygiene and Tropical Medicine and externally by University of Zimbabwe Research Support Centre in Zimbabwe and Malawi-Liverpool-Wellcome Trust Clinical Research Programme clinical trials unit in Malawi. |
| Ethics and dissemination | | |
| Research ethics approval | 24 | Ethics approval was obtained locally from the College of Medicine Research Ethics Committee (COMREC) in Malawi (approval number P.04/15/1719), the Harare Central Hospital Ethics Committee (Does not have a reference number) and the Medical Research Council of Zimbabwe (approval number MRCZ/A/1946) and from the London School of Hygiene & Tropical Medicine Ethics Committee (approval number 8818). |
| Protocol amendments | 25 | Important protocol modifications (eg, changes to eligibility criteria, outcomes, analyses) will be communicated first to IRBs for approval before implementation. |
| Consent or assent | 26a | Research nurses will obtain informed consent and informed consent and assent in case of participants < 18 years. All trial participants will give written or witnessed (with thumb print for illiterate participants) consent before undergoing any trial procedures. |
|  | 26b | Additional consent provisions for collection and use of participant data and biological specimens in ancillary studies (**not applicable**). |
| Confidentiality | 27 | Only authorised personnel will handle the study data with password protection of both the computer and the study database. Final data will be fully anonymised to remove any participant identifying information to uphold confidentiality. |
| Declaration of interests | 28 | All principal investigators declare no other competing interests for the overall trial and each study site. |
| Access to data | 29 | All trial investigators and DSMB members will have access to the final trial dataset. There are no contractual agreements that limit access for investigators. The final fully anonymised data from the study will be made publicly available through the LSHTM data repository (<http://datacompass.lshtm.ac.uk/>. |
| Ancillary and post-trial care | 30 | There are no ancillary and post-trial care. The study is covered by no-fault insurance policy for compensation to those who suffer harm during trial participation. |
| Dissemination policy | 31a | Findings will also be presented at peer-reviewed regional and international conferences. |
|  | 31b | ICMJE authorship eligibility guidelines will be followed during publication. |
|  | 31c | The final fully anonymised data from the study will be made publicly available through the LSHTM data repository (<http://datacompass.lshtm.ac.uk/>. |
| Appendices |  |  |
| Informed consent materials | 32 | Model consent form and other related documentation given to participants and authorised surrogates (See attached Information sheet and consent form) |
| Biological specimens | 33 | Plans for collection, laboratory evaluation, and storage of biological specimens for genetic or molecular analysis in the current trial and for future use in ancillary studies, (**Not applicable**) |

*It is strongly recommended that this checklist be read in conjunction with the SPIRIT 2013 Explanation & Elaboration for important clarification on the items. Amendments to the protocol should be tracked and dated. The SPIRIT checklist is copyrighted by the SPIRIT Group under the Creative Commons “[Attribution-NonCommercial-NoDerivs 3.0 Unported](http://www.creativecommons.org/licenses/by-nc-nd/3.0/)” license.
